# 11-Year Trends in Pregnancy-Related Health Indicators in Maine, 2000–2010

**DOI:** 10.1155/2014/780626

**Published:** 2014-11-13

**Authors:** David E. Harris, AbouEl-Makarim Aboueissa, Nancy Baugh, Cheryl Sarton, Erika Lichter

**Affiliations:** ^1^School of Nursing, University of Southern Maine, Portland, ME 04104, USA; ^2^Department of Mathematics and Statistics, University of Southern Maine, Portland, ME 04104, USA; ^3^Department of Applied Medical Sciences, University of Southern Maine, Portland, ME 04104, USA

## Abstract

The objective of this study is to understand health and demographic trends among mothers and infants in Maine relative to the goals of *Healthy People 2020.* Pregnancy risk assessment monitoring system (PRAMS) data from Maine for 2000–2010 were used to determine yearly values of pregnancy-related variables. Means (for continuous variables) and percentages (for categorical variables) were calculated using the survey procedures in SAS. Linear trend analysis was applied with study year as the independent variable. The slope and significance of the trend were then calculated. Over the study period, new mothers in Maine became better educated but the fraction of households with incomes <$20,000/year remained stagnant. Maternal prepregnancy BMI increased. Average pregnancy weight gain decreased but the number of women whose pregnancy weight gain was within the recommended range was unchanged. The rates of smoking and alcohol consumption (before and during pregnancy) increased. The Caesarean section rate rose and the fraction of infants born premature (<37 wks gestation) or underweight (<2500 gms) remained unchanged. The fraction of infants who were breast-fed increased. These results suggest that, despite some positive trends, Maine faces significant challenges in meeting *Healthy People 2020* goals.

## 1. Introduction

Women's health and health behaviors before, during, and after pregnancy can impact the course and outcome of their pregnancy as well as the health of the children born from those pregnancies. Maternal smoking before [1], during [2, 3], and after [4] pregnancy is a risk to children's health and development. Maternal smoking [5] and even moderate drinking [6] during pregnancy increase the risk of having a small for gestational age infant which could prolong hospital stays, require admission to a neonatal intensive care unit, increase mortality during infancy [7], and produce developmental problems as the child grows [8]. Maternal prepregnancy obesity and excessive weight gain during pregnancy are associated with increased risk of pregnancy complications and childhood health challenges [9–11] while inadequate gestational weight gain is associated with low birth weight [10].

The presence of two parents in a family unit and adequate family income can also impact child health. Poverty is associated with health challenges while families with incomes near or below the federal poverty level and single-parent households are at risk for food insecurity [12] which is, in turn, a health risk for children [13, 14]. There are also maternal behaviors that can improve infant health. Seeking early prenatal care is associated with reduced risk of having a low birth weight infant and of infant death [15]. Breast-feeding an infant is associated with reduced rates of childhood illnesses [16], improved cognitive development at school age, and health benefits that last into adulthood [17].

When the US Department of Health and Human Services led an interagency workgroup known as* Healthy People 2020 *in the development of national health objectives, they specified multiple maternal and infant health objectives. These include the objectives of reducing the number of women who smoke and drink alcohol (before, during, and after pregnancy), increasing the number of women who enter pregnancy at a healthy weight and have a healthy level of weight gain during pregnancy, increasing the number of women who receive early prenatal care, decreasing the number of infants who are born by Caesarean section, premature, or at low birth weight, and increasing the number of infants who are breast-fed [18].* Healthy People 2020* also recognizes the negative impact that poverty can have on the health, as well as the high rate of poverty for children nationwide [18].

This study analyzes 11 years of data (2000–2010) from the pregnancy risk assessment monitoring system (PRAMS) [19] for the state of Maine. PRAMS, a joint effort of the US Center for Disease Control and Prevention and Individual State Departments of Health, is a public health survey that uses standardized collection techniques to gather information from women who have recently delivered live babies. PRAMS data provide a powerful tool for analyzing either a small number of pregnancy-related variables across broad geographical regions or a broad range of pregnancy-related variables with a more limited geographical context. By focusing on the state of Maine, this study takes the second approach.

The objective of this study is to define the year-to-year trends in health variables for women giving birth in Maine and for their infants. These results will indicate if Maine is likely to meet* Healthy People 2020* maternal and infant health objectives. This information is important because, while Maine has relatively low rates of infant mortality and low birth weight compared to national averages, it is a state where these important indicators are not improving [20]. The results of this study may suggest interventions for improving maternal-child health in Maine. Maine is also a state with communities distributed across the rural-urban continuum and where rurality impacts health [21]. Thus, the results of this study may also be of interest to those who work in similar areas. Finally, these results will also be of interest to anyone who wishes to use PRAMS data to analyze trends in their own area relative to Maine or to* Healthy People 2020 *guidelines [18].

## 2. Methods

PRAMS identifies women who gave birth to a live infant within the previous 2–4 months from birth certificate data. It then uses mailed questionnaires and telephone follow-up to obtain information from a stratified representative sample of these women, with members of high-risk groups oversampled, and links questionnaire answers to birth certificate data [21]. In Maine, as elsewhere, women with low birth weight infants are oversampled. PRAMS data for Maine from 2000 to 2010 were obtained from the Maine Center for Disease Control and Prevention [22].

For this study, variables from multiple categories were analyzed.

- Maternal demographic and prepregnancy health indicator variables analyzed included (1) age, (2) marital status as a dichotomous variable, (3) household income (converted to a dichotomous variable of <$20,000 or >$20,000/year because PRAMS used multiple questionnaire formats with different income cut points during the study period but all versions had a cut point at $20,000/year), (4) education as a dichotomous variable (≤12 yrs or >12 yrs), (5) race as a dichotomous variable (white versus “other”) reflecting the low level of racial diversity in Maine, and (6) age and % of women with no previous live births.
- Maternal weight and pregnancy weight gain variables analyzed included (1) maternal prepregnancy height and weight (used to calculate BMI) and (2) weight gain during pregnancy as a continuous variable, as a categorical variable (<15 lbs, 15–45 lbs, and >45 lbs), and as a categorical variable relative to current weight gain recommendations (< recommended range, within recommended range, and > recommended range).
- Prenatal care variables analyzed included (1) gestational age at earliest prenatal care in weeks as a continuous variable and (2) the fraction of women who received their first prenatal care within the first trimester (≤12 weeks).
- Maternal tobacco and alcohol consumption variables analyzed included (1) alcohol consumption in the 3 months before pregnancy and in the last 3 months of pregnancy as dichotomous variables and (2) smoking in the 3 months before pregnancy, in the last 3 months of pregnancy, and at the time the questionnaire was administered, all as dichotomous variables.
- Variables related to Caesarean section birth analyzed included (1) the total rate of Caesarean section birth as well as (2) the rate of first-time and (3) the rate of repeat Caesarean section birth.
- Infant outcomes variables analyzed included (1) the rate of plural births, (2) the fraction of infants born at gestational age <37 weeks, (3) the fraction of infants admitted to an intensive care unit, (4) the length of hospital stay as a categorical variable (1-2 days, 3–5 days, and ≥6 days), and (5) birth weights both as a continuous variable and as a categorical variable (<2500 gms, 2500–3999 gms, and ≥4000 gms).
- Variables related to breast-feeding analyzed included the fraction of women who (1) never breast-fed their infants, (2) breast-fed for <8 weeks, and (3) breast-fed for ≥8 weeks.

Infant birth weight was obtained from the birth certificate; all other variables were self-reported. All results reflect values only among those who took the PRAMS survey.

Three different forms of the PRAMS questionnaire were used during the time period covered by this study (2000–2010). The Phase 4 questionnaire was in use until 2003, the Phase 5 questionnaire was used from 2004 to 2008, and the Phase 6 questionnaire was used from 2009 onward. It is important to note that there were minor changes in the format of PRAMS questions about smoking and drinking over the time covered by this study. For smoking, PRAMS asks a screening question to determine if a study participant smoked cigarettes and follows up with specific questions about smoking before, during, and after pregnancy only if the subject answers the screening question in the affirmative. However, PRAMS Phases 4 and 5 used “Have you smoked at least 100 cigarettes in the past 2 years?” as a screening question while Phase 6 uses “Have you smoked any cigarettes in the past 2 years?” [23]. In the case of the amount of alcohol consumption in the 3 months before pregnancy, the Phase 4 questionnaire allowed a response of “I don't know” while Phases 5 and 6 did not [23]. This necessitates that temporal trends in preconception smoking and alcohol consumption variables be interpreted with caution.

Results were analyzed using the survey procedures in SAS to adjust for the complex sampling strategy of the PRAMS dataset. The PRAMS dataset contains weighting variables, including the weighting stratum and the weighting coefficient, for each entry. This allows the statistical analysis software package used (SAS) to adjust for the complex sampling strategy of PRAMS (oversampling) and effectively “undo” the impact of oversampling. This produces results that accurately reflect the full population from which the PRAMS dataset was obtained and still take advantage of the reduced “noise” that oversampling is designed to produce.

Means (for continuous variables) and percentages (for categorical variables) were calculated for the overall study period and for each study year, along with 95% confidence intervals. Significant differences between years were tested for using *F*-tests (for continuous variables) and Chi-square tests (for dichotomous variables). If significant differences between years existed, linear trend analysis was applied with study year as the independent variable. The slope and significance of the trend were then calculated. Significance was accepted at *P* < 0.05.

## 3. Results

During the 11-year study period, Maine PRAMS questionnaires were obtained from 12,600 women, an average of 1,145.5/year. The PRAMS methodology has a minimum overall response rate threshold policy for the release of data of 70% for data prior to 2007 and 65% for data from 2007 on. The questionnaire response rate in Maine is consistently well above 70% but did not vary significantly over the study period. We excluded 29 participants for whom infant birth weight was unknown, leaving 12,571 possible respondents to any question. For each question, all the responses that were available were analyzed. The number of responses for each variable was consistently >95% of the possible respondents.

**Figure 1:**
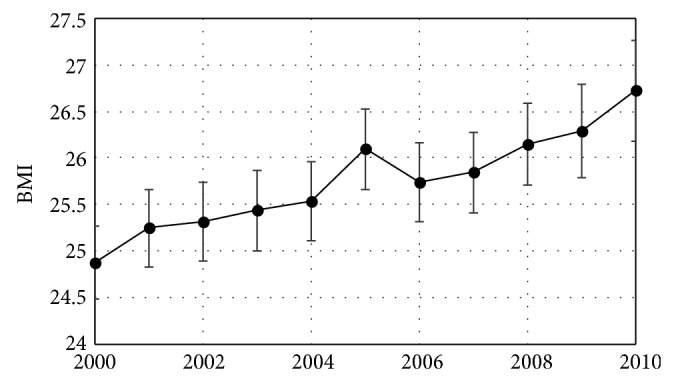
Maternal prepregnancy BMI ± 95% CI of women giving birth in Maine shown by year. Mean BMI increased at an average rate of 0.15 BMI units/year.* Healthy People 2020* objectives include a 10% increase in the proportion of women who had a healthy weight prior to pregnancy. Given that BMI > 25 is currently defined as overweight while BMI > 30 is currently defined as obese, Maine is not moving toward this goal.

The average age of women giving birth in Maine during the study period was 28.1 years and the average age of women giving birth to their first child was 26.1 years. Over the study period, 31.8% of new mothers were in households with incomes less than $20,000 per year. There were no significant trends over time in these variables (Table 1). There were significant trends in other prepregnancy health and demographic variables. Average maternal BMI was 24.9 in 2000 and increased by 0.15 BMI units/year during the study period (Table 1, Figure 1). In 2000, 69.9% of women giving birth in Maine were married (decreasing by 0.06%/year during the study period), 45.9% were having their first child (increasing by 0.01% per year during the study period), 49.9% had no education past high school (decreasing at 0.02% per year), and 2.8% reported a race other than white (increasing at 0.05% per year) (Table 1).

Average maternal weight gain during pregnancy was 31.3 lbs in 2000 and fell by 0.24 lbs/year during the study period. This change resulted from an increase in the fraction of women who gained <15 lbs and a decrease in the number of women who gained 15–45 lbs. The fraction who gained >45 lbs did not change. The Institute of Medicine (IOM) defines healthy levels of maternal weight gain inversely with obesity status. For instance, the IOM recommends that women who have a normal prepregnancy weight gain 25–35 lbs during pregnancy while those who are overweight prior to conception gain only 15–25 lbs [24]. By IOM definitions, 36.4% of pregnant Maine women had gestational weight gain within the recommended range, 21.1% gained less than the recommended amount, and 42.4% gained more than the recommended amount of weight. Only the percent gaining less than the recommended amount showed a significant temporal trend; it increased by 0.4%/year over the study period.

**Figure 2:**
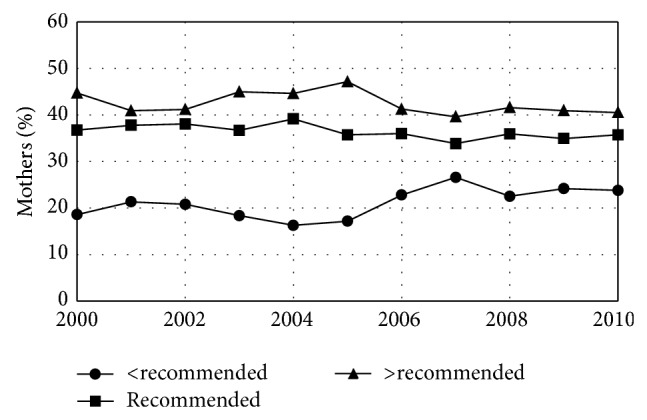
Gestational weight gain for women giving birth in Maine by year. The fraction of women gaining less than the recommended amount of weight increased while the fraction gaining an amount of weight that was within the recommended range or above that range remained unchanged.* Healthy People 2020 *objectives include an increase in the proportion of women who achieved recommended levels of weight gain during pregnancy (numerical goal under development). Maine is not moving toward this goal.

The mean time at which women received their first prenatal care was 8.6 weeks and the average fraction who received prenatal care in the first trimester was 92.8%. There were no significant trends in either of these variables during the study period (Figure 2, Table 2).

**Figure 3:**
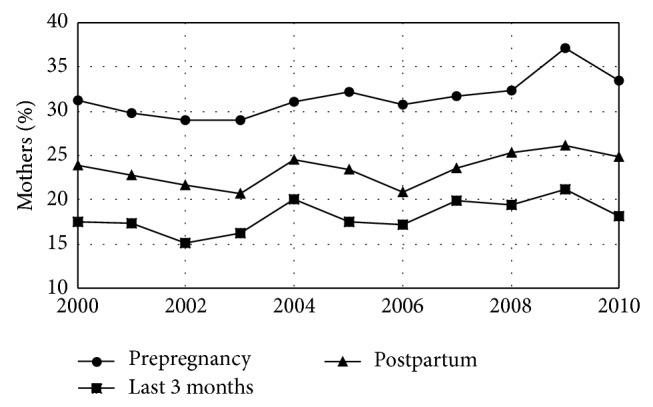
Maternal smoking by year. Fraction of mothers who smoked cigarettes in the last 3 months before pregnancy, the last 3 months of pregnancy, and postpartum (at the time of the questionnaire) for women giving birth in Maine by year.* Healthy People 2020* has objectives of a 10% increase in the percent of women who did not smoke cigarettes prior to pregnancy as well as a 10% increase in abstinence from cigarettes among pregnant women. Maine is not moving toward this goal.

**Figure 4:**
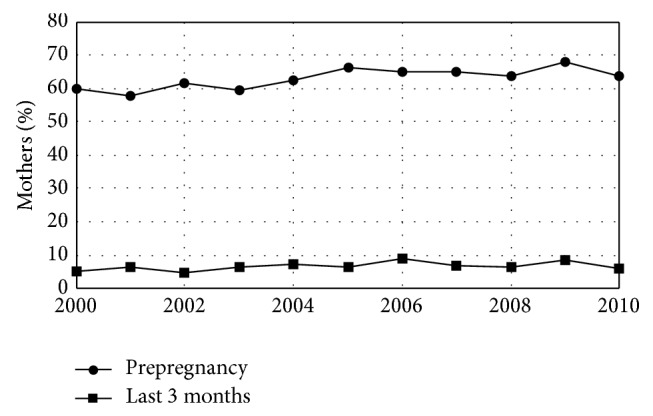
Maternal alcohol consumption by year. Fraction of mothers who drank alcohol in the last 3 months before pregnancy and the last 3 months of pregnancy for women giving birth in Maine by year.* Healthy People 2020* has objectives of a 10% increase in the percent of women who did not drink alcohol prior to pregnancy as well as a 10% increase in abstinence from alcohol among pregnant women. Maine is not moving toward this goal.

**Figure 5:**
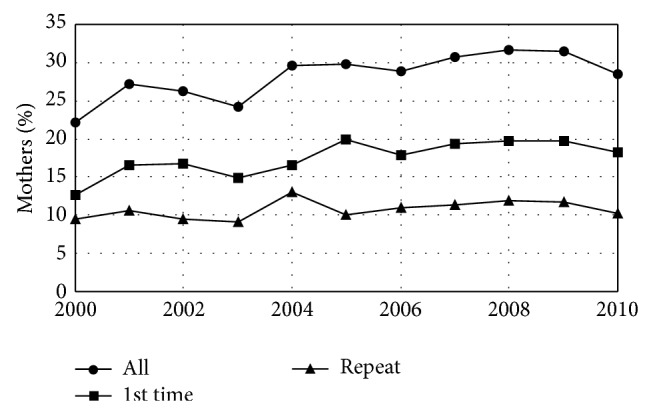
Caesarean section rate by year. Fraction of mothers giving birth in Maine who delivered by Caesarean section by year. The overall Caesarean section rate increased as a result of an increase in the rate of first-time Caesarean sections. The rate of repeat Caesarean sections remained unchanged.* Healthy People 2020* has the objective of a 10% reduction in the rate of births by first-time Caesarean section. Maine is not moving toward this goal.

In 2000, 31.3% of women in this study smoked cigarettes in the 3 months prior to pregnancy (increasing by 0.02% per year during the study period), and 60.1% drank alcohol in the 3 months prior to pregnancy (increasing by 0.03% per year during the study period). The fractions of pregnant women who smoked and drank during the last 3 months of pregnancy also increased during the study period at similar rates but the fraction who smoked at the time of the questionnaire did not change (Figures 3 and 4, Table 3). The overall rate of Caesarean section deliveries was 22.2% in 2000 and increased at an average rate of 0.04%/year during the study period. This increase was the result of an increase in first-time Caesarean sections which started at 12.7% in 2000 and also increased by an average of 0.04%/year. The rate of repeat Caesarean sections did not change (Figure 5, Table 4).

**Figure 6:**
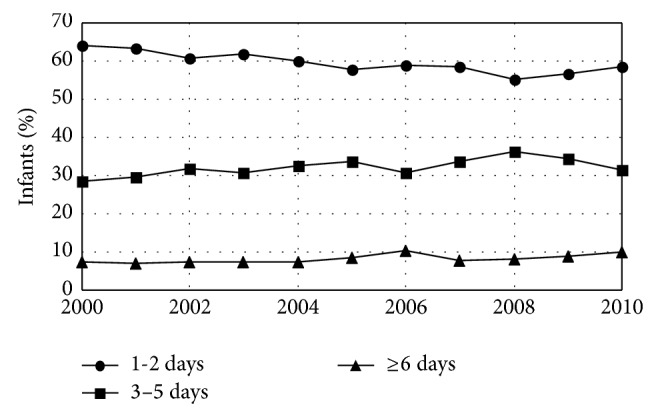
Hospital length of stay for infants born in Maine hospitals by year. The fraction of infants who were hospitalized for 1-2 days after birth declined while the fraction hospitalized in both of the two longer stay categories increased.

**Figure 7:**
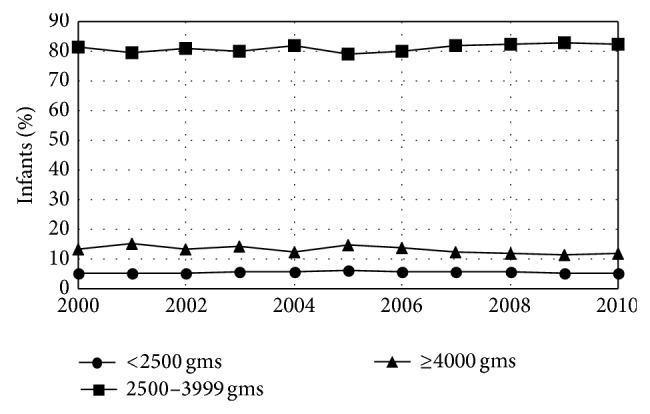
Birth weights for infants born in Maine by year. The fraction of infants who weighed ≥4000 gms at birth declined over time.* Healthy People 2020* has the objective of a reduction in low birth weight births to 7.8% of total births. Maine currently meets this goal.

**Figure 8:**
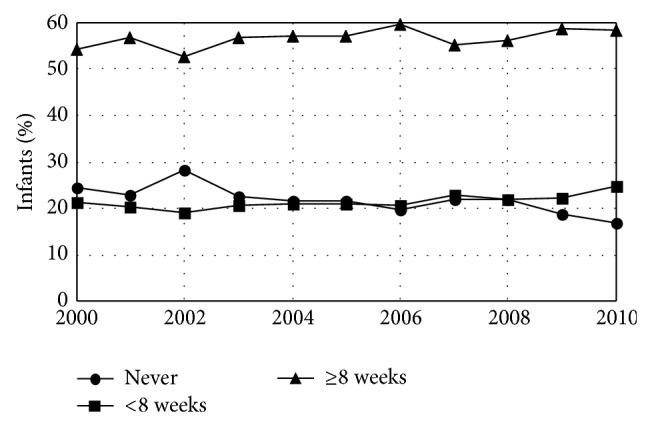
Fraction of infants breast-fed in Maine by year. The fraction never breast-fed declined while the fractions beast-fed for <8 wks and ≥8 wks increased.* Healthy People 2020* has the objective of increasing the number of infants ever breast-fed to 81.9%. Maine does not currently meet this goal but is moving toward it.

There were no significant trends during the study period in a range of infant outcome variables including the fraction of plural births (1.5%), the fraction of births that were premature (<37 weeks gestation) (8.1%), and the fraction of infants admitted to an intensive care unit (9.3%) (Table 5). However, there were significant trends in the length of time infants spent in the hospital after birth with fewer staying 1-2 days and more staying either 3–5 days or longer (Figure 6, Table 6). There were also significant trends in infant weight. Average infant weight was 3416 gms in 2000 and fell by 4.4 gms/year. This decline resulted from fewer infants with birth weights >4000 (a cutoff that has been used for newborn macrosomia [25]) with no change in the fraction born <2500 grams (a weight well below the 3rd percentile for both male and female infants [26]) (Figure 7, Table 7). There were also significant trends in breast-feeding. The fraction of infants who were never breast-fed declined by 0.04%/year while the fraction who were breast-fed for <8 weeks increased by 0.02%/year and the fraction who were breast-fed for ≥8 weeks increased by 0.01%/year. Overall, 78.2% of infants born during the study period were breast-fed at least some and 56.7% were breast-fed for ≥8 weeks (Figure 8, Table 8).

## 4. Discussion 

### 4.1. Demographics

The demographic results reported in Table 1 show increases during the study period in the fraction of women giving birth in Maine who had education past high school, the fraction who reported a race other than white, the fraction who were unmarried, the fraction who were giving birth to their first baby, and the age of first-time (but not all) mothers. The educational trend is not unexpected. Maine high school graduation rates are rising [27], so more Mainers are eligible to pursue postsecondary education. This trend toward better educated mothers is positive. More extensive education is associated with improved health [28] although the effect may be via the increased income that comes with more education [29].

The declining marriage rate found in this study follows national trends at work since the 1960s [30]. However, it too has health implications. Being unmarried is generally associated with poorer health [31] and parental health has an impact on children's health [32]. Furthermore, children born to unmarried women are at higher risk of adverse birth outcomes including low birth weight, preterm birth, and infant mortality than are children born to married women [33], probably because being an unmarried mother is a marker for having a low income and a risk factor for a range of measures of social disadvantage including food insecurity [12]. The rise in the number of women giving birth to their first child and the increasing age of first time mothers may suggest delayed childbearing, also a nationwide trend [34], while the increase in racial diversity reported here suggests that Maine, like the country as a whole, is becoming more racially diverse.

One troubling finding reported here is that the fraction of women giving birth in Maine with annual household incomes less than $20,000 has remained constant over the 11-year study period (Table 1) even as income poverty thresholds have risen. A $20,000/year income represented 141% of the federal poverty limit for a family of 3 in 2000 but only 109% of the poverty level in 2010 and 102% of the federal poverty limit for a family of 3 in 2013 [35]. This suggests that more Maine children may have been born into households challenged by poverty as the study period progressed, although more work is needed to determine this. This may represent a health challenge because low income is correlated with higher rates of prepregnancy smoking, obesity, and chronic health challenges [36] and because poverty is associated with increased risk of complications during pregnancy [37].* Healthy People 2020* recognizes the negative impact of childhood poverty on health but sets no specific objectives in the area of childhood poverty [18].

### 4.2. Obesity and Gestational Weight Gain

Prepregnancy obesity is associated with an increased incidence of gestational diabetes, gestational hypertension, preeclampsia, Caesarean section [11, 38], macrosomia, postpartum hemorrhage, congenital defects, miscarriage, stillbirth, maternal mortality [39], and childhood obesity [11]. These impacts are generally exacerbated by excessive weight gain during pregnancy [9, 10] while inadequate gestational weight gain is associated with low birth weight [10]. As a result,* Healthy People 2020 *objectives include a 10% increase in the proportion of women who had a healthy weight prior to pregnancy and an increase in the proportion of women who achieved recommended levels of weight gain during pregnancy (numerical goal under development) [18]. Evidence from the National Health and Nutrition Examination Survey (NHANES) show that obesity rates among US adults may have plateaued, although at an unacceptably high level [40]. However, PRAMS results suggest that preconception obesity rates continued to increase nationally, at least through 2009, and that the number of women who had a healthy weight prior to pregnancy was just over 50%. Although obesity rates are similar in Maine to national levels, the trends in obesity in Maine are less clear, with preconception obesity rates increasing significantly only for those with BMI ≥40 in a comparison of 2003 versus 2006 versus 2009 [41].

This study found that the average preconception BMI of Maine mothers for the entire study period (25.8) was in the overweight range (25–29.9) [42] and that the yearly average BMI increased steadily from 2000 to 2010, reaching 26.7 by 2010 (Table 1, Figure 1). Because this study used a continuous variable (BMI) rather than a categorical variable with somewhat arbitrary cut points [42] that can change over time [43] and for which age may need to be considered in younger women [41] (obesity rates), these results give a clear picture of increasing preconception weight in Maine. This study also found that while gestational weight gain is declining in Maine, this change results from an increase in the fraction of women who gained less than the IOM recommended amount of weight with no change in the number whose weight gain was within the recommended range (Figure 2, Table 2).

Helping women achieve a healthy level of weight gain during pregnancy is not easy but it is possible. Simply having a practitioner give pregnant women advice about healthy weight gain during a standard prenatal visit has little impact on whether or not a women actually achieves healthy weight gain [44]. However, a light to moderate intensity exercise program for pregnant women can prevent excessive gestational weight gain [45]. Postpartum weight retention is also a health issue. One face-to-face meeting during pregnancy with a designated interventionist with telephonic and mail follow-up focusing on healthy diet, increased exercise, and self-monitoring of eating, exercise, and weight gain was found to decrease weight retention 12 months postpartum but not to increase the number of women who regained their prepregnancy weight [46]. Thus, interventions that go beyond what is possible at a prenatal visit and include active participation by the patients are probably needed for Maine to reverse current trends of increasing prepregnancy BMI with no increase in the number of women who achieved healthy weight gain during pregnancy as will be necessary if Maine is to meet* Healthy People 2020 *goals around prepregnancy weight and gestational weight gain [18].

### 4.3. Tobacco and Alcohol

The high rates of smoking and drinking reported here also have negative health implications. Smoking during pregnancy increases the risks of pregnancy complications including spontaneous abortions, ectopic pregnancies, and placenta previa. It may also increase the risk that the child born from that pregnancy will experience behavioral disorders [47]. Heavy smoking before pregnancy is associated with children having lower cognitive abilities even if the mother has quit smoking before she conceives [1]. Alcohol consumption in the months prior to pregnancy is also generally considered a risk to the child born from the subsequent pregnancy. Heavy drinking in the 3 months prior to conception is associated with low birth weight [6] and alcohol consumption prior to pregnancy may interact with smoking during that same period to produce a particularly high risk of cardiac defect [48]. However, not all studies find an association between moderate alcohol consumption early in pregnancy and negative outcomes such as low birth weight, preeclampsia, and preterm birth [49].

Following the belief that both maternal smoking and drinking are health risks to a developing fetus,* Healthy People 2020* has objectives of a 10% increase in the percent of women who did not smoke cigarettes or drink alcohol prior to pregnancy as well as a 10% increase in abstinence from alcohol and cigarettes among pregnant women [18]. For the entire study period, the results reported here show a preconception nonsmoking rate for Maine of 68.4% and a preconception nondrinking rate of 36.9%. During pregnancy, 81.9% of expectant women did not smoke and 93.3% did not drink (Figures 3 and 4, Table 3). The smoking results extend previous reports for shorter time periods [50, 51]. Because of minor changes in the PRAMS questions around smoking and drinking during the study period, the significant increases in preconception smoking and drinking found here may reflect the change in PRAMS methodology rather than an actual increase and must be interpreted with caution. A multisite study of smoking that included Maine included the change in smoking question in its analysis and found no significant increase in smoking prior to pregnancy [52]. However, smoking levels in Maine reported here are more than double* Health People 2020 *goals prior to pregnancy and over 15 times national goals during pregnancy. Drinking levels in Maine are 40% higher than national goals prior to pregnancy and are nearly 4 times the* Healthy People 2020* goals during pregnancy. (compare Table 3 to [18].) Furthermore, there is no sign, either in the results reported here or in previously reported results, that smoking and drinking before or during pregnancy are declining in Maine as would be necessary to meet* Healthy People 2020* goals.

As with weight gain, a single intervention during a prenatal visit may not be enough to positively impact smoking and drinking behavior. A brief computer-based intervention during a prenatal visit failed to reduce drinking during pregnancy [53] but counselling combined with incentives, feedback, and peer support did prove effective at getting pregnant women who smoked to quit [54]. Even prepregnancy behavior is amenable to change through robust interventions. Motivational interviewing and feedback have been shown to reduce alcohol-exposed pregnancy risk among nonpregnant college students [55]. Clearly, major efforts will be necessary for Maine to reach the goals of* Healthy People 2020* for reducing pregnancy-related smoking and alcohol consumption. As is the case with weight gain, interventions that go beyond what is possible at a prenatal visit and include active participation by the patients are probably needed.

### 4.4. Prenatal Care

Seeking prenatal care is associated with reduced risk of delivering a low birth weight infant and of infant death [56] and* Healthy People 2020 *has an objective of increasing the percent of women who received prenatal care beginning in the first trimester by 10%. The results reported here show that 92.8% of pregnant women in Maine obtain prenatal care within the first trimmest. However, they here fail to show any change in the fraction of women who access early prenatal care (Table 2). Community outreach and education may be necessary to reverse this trend.

### 4.5. Birth and Postpartum

Preterm birth (birth prior to 37 weeks gestation) is a leading cause of respiratory and neurological disability in infants and infant death [57, 58]. Low birth weight/small for gestational age infants (generally those <2500 gms in weight) are also at risk for increased mortality [59] as well as problems around thermoregulation, hypoglycemia, and sepsis [60, 61]. Birth by Caesarean section subjects the mother to major abdominal surgery and is a risk for reduced subsequent fertility [62]. Although randomized controlled studies are lacking, Caesarean section birth may also place infants at risk for several health challenges including obesity, metabolic syndrome, hypertension, type 1 diabetes, asthma, and inflammatory bowel disease [63] probably because babies born by Caesarean section do not experience the physiological stress of labor and vaginal birth [64].

*Healthy People 2020* has multiple specific objectives for improved birth and postpartum outcomes including 10% reductions in preterm birth rate and the rate of births by first-time Caesarean section and a reduction in low birth weight births to 7.8% of total births [18]. The results reported here show that the rate of first-time Caesarean sections in Maine is increasing (Figure 5, Table 4) and show no decrease in the rate of preterm births (Table 5). Low weight (<2500 gms) births in Maine were within the* Healthy People 2020* objectives but were not decreasing (Figure 7, Table 7). Caesarean section rates, at least, may be amendable to nonclinical intervention. Both a nurse-led relaxation program and guideline implementation programs with mandatory second opinion have been shown to reduce Caesarean section rates [65].

Breast-feeding an infant is associated with a reduction in the risk of ear, respiratory, and skin diseases; GI diseases of infancy including nonspecific gastroenteritis and necrotizing enterocolitis; metabolic diseases including obesity, type 1 diabetes, and type 2 diabetes; childhood leukemia; and sudden infant death syndrome (SIDS) [16]. It is also associated with improved cognitive development at school age; lower blood pressure persisting into adulthood; and lower risk of hypercholesterolemia, obesity, and type 2 diabetes mellitus among adults who were breast-fed as infants [17].

The fraction of Maine babies ever breast-fed was 78.1%, 3.8% below* Healthy People 2020 *objectives [18], but rate of breast-feeding was increasing (Figure 8, Table 8). It may be possible to further improve this rate by some simple interventions. A brief questionnaire that explores a baby's nursing behavior as a neonate has proven effective at predicting successful nursing behavior at 3 to 6 months of age [66]. This raises the possibility that infants who may not succeed at longer-term breast-feeding can be identified early and their mothers provided extra support. There is also evidence that home visits which combine education and patient-specific advice beginning before a new mother returns to work and continuing after she begins working reduce anxiety and increase the frequency of breast-feeding among working mothers in Turkey [67]. Once a mother has returned to work, policies that encourage women to nurse and/or pump breast milk in the workplace combined with coworker encouragement are associated with breast-feeding past 6 months after return to work in Taiwan [68]. These findings highlight the importance of policies and interventions that continue to support new mothers in breast feeding after birth.

## 5. Limitation and Conclusions

The PRAMS dataset is a rich source of information but working with it comes with limitations. First, as discussed in the matter of smoking and drinking variables, changes in the question format were introduced during the study period. Although minor, these changes probably account for the increasing trend we found in prepregnancy smoking (compare Tong et al., 2013 [52], to Table 3). Nonetheless, there is no indication that prepregnancy smoking rates are declining in Maine, so the conclusion presented here that much more needs to be done in this area to meet* Healthy People 2020 *objectives is valid.

Second,* Healthy People 2020 *does not use PRAMS as a data source, so it can be difficult to compare absolute measures from PRAMS data in this study to* Healthy People 2020 *objectives. For instance, in the matter of breast-feeding,* Healthy People 2020 *uses results from the National Immunization Survey (NIS) which uses telephone interviews generated from a randomized list of phone numbers to locate households with young children [69] rather than the PRAMS approach of beginning with birth certificate contact information. Thus, the most meaningful comparisons between the results reported here and* Healthy People 2020 *objectives may be in trends, and that has been the main focus of the analysis presented here. Fortunately, many of the objectives of* Healthy People 2020 *are presented as % changes. There are some examples, however, such as smoking rates and drinking rates, where Maine PRAMS results are far below* Healthy People 2020 *objectives. These almost certainly represent areas where Maine needs to improve.

In summary, this study identifies prepregnancy, prenatal, and postpartum demographic, behavioral, and health trends for women having children in Maine from 2000 to 2010 and for their babies which may challenge Maine's efforts to meet* Healthy People 2020* objectives. These results may suggest specific health priorities and interventions for Maine and areas of important inquiry for those in other states.

## Figures and Tables

**Table 1:** Prepregnancy demographics for women giving birth in Maine, 2000–2010.

Variable	*N*	Grand mean	95% CI		*P* trend	Slope
Maternal age (yrs)	12561	28.1	28.0	28.3	0.351	
Maternal age 1st birth (yrs)	6124	26.1	26.0	26.3	0.804	
Maternal BMI	12561	25.8	25.6	25.9	**<0.0001**	0.153
No previous live birth (%)	12460	45.7	44.7	46.8	**0.042**	0.014
Married (%)	12561	63.6	62.5	64.6	**<0.0001**	−0.062
Maternal education ≤12 yrs (%)	12514	45.2	44.2	46.2	**0.0009**	−0.023
Household income <$20 k/yr (%)	11981	31.8	30.8	32.8	0.210	
Maternal race not white (%)	12270	3.1	2.7	3.5	**0.020**	0.049

**Table 2:** Pregnancy weight gain and prenatal care for women giving birth in Maine, 2000–2010.

Variable	Grand mean	95% CI		*P* trend	Slope
Weight gain (lbs)	29.7	29.4	30.0	**<0.0001**	**−0.24**
<15 lbs (%)	11.2	10.5	11.8	**<0.0001**	**0.07**
15–45 lbs (%)	77.9	77.0	78.8	**<0.0001**	**−0.04**
>45 lbs (%)	10.9	10.3	11.6	0.83	
<recommended range (%)	21.1	20.3	22.0	**<0.0001**	**0.04**
Within recommended range (%)	36.4	35.4	37.4	0.07	
>recommended range (%)	42.5	41.5	43.5	0.07	
1st prenatal care (weeks)	8.6	8.5	8.7	0.65	
Prenatal care 1st trimester (%)	92.8	92.2	93.4	0.47	

**Table 3:** Alcohol consumption and smoking by women giving birth in Maine, 2000–2010.

Variable	*N*	Grand mean	95% CI		*P* trend	Slope
Drank 3 mths before pregnancy (%)	12311	63.1	62.1	64.1	**<0.0001**	0.031
Drank last 3 mths of pregnancy (%)	12388	6.7	6.2	7.2	**0.02**	0.03
Smoked 3 mths before pregnancy (%)	12371	31.6	30.6	32.6	**0.002**	0.024
Smoked last 3 mths of pregnancy (%)	12429	18.1	17.3	19.0	**0.01**	0.02
Mother currently smokes (%)	12440	23.4	22.5	24.3	0.06	

**Table 4:** Caesarean section rates for women giving birth in Maine, 2000–2010.

Variable	Grand mean	95% CI		*P* trend	Slope
All C-sections (%)	28.2	27.3	29.1	**<0.0001**	**0.035**
First-time C-sections (%)	17.5	16.7	18.3	**<0.0001**	**0.038**
Repeat C-sections (%)	10.7	10.1	11.4	0.12	

**Table 5:** Infant outcomes for newborns in Maine, 2000–2010.

Variable	*N*	Grand mean	95% CI		*P* trend	Slope
Plural births (%)	12561	1.5	1.3	1.7	0.60	
Gestational age <37 wks (%)	12556	8.1	7.6	8.5	0.37	
Infant admitted to ICU (%)	12479	9.3	8.8	9.8	0.05	

**Table 6:** Length of hospital stay for infants born in Maine hospitals, 2000–2010.

Variable	Grand mean	95% CI		*P* trend	Slope
1-2 days (%)	59.6	58.6	60.6	**<0.0001**	**−0.03**
3–5 days (%)	32.2	31.2	33.2	**0.004**	**0.02**
≥6 days (%)	8.2	7.7	8.7	**0.002**	**0.03**

**Table 7:** Birth weight distribution for infants born in Maine, 2000–2010.

Variable	Grand mean	95% CI		*P* trend	Slope
Infant birth weight (gms)	3409.1	3399.3	3418.8	**0.01**	**−4.4**
Birth wt <2500 gms (%)	5.7	5.6	5.7	0.27	
Birth wt 2500–3999 gms (%)	81.1	80.4	81.8	0.05	
Birth wt ≥4000 gms (%)	13.2	13.5	13.9	**0.02**	**−0.02**

**Table 8:** Breast-feeding by women giving birth in Maine, 2000–2010.

Variable	Grand mean	95% CI		*P* trend	Slope
Never breast-fed (%)	21.9	21.0	22.8	**<0.0001**	**−0.04**
Breast-fed <8 wks (%)	21.5	20.6	22.3	**0.02**	**0.02**
Breast-fed ≥8 wks (%)	56.7	55.6	57.7	**0.04**	**0.01**
